# Crabs Mediate Interactions between Native and Invasive Salt Marsh Plants: A Mesocosm Study

**DOI:** 10.1371/journal.pone.0074095

**Published:** 2013-09-04

**Authors:** Xiao-dong Zhang, Xin Jia, Yang-yun Chen, Jun-jiong Shao, Xin-ru Wu, Lei Shang, Bo Li

**Affiliations:** 1 Coastal Ecosystems Research Station of the Yangtze River Estuary, Ministry of Education Key Laboratory for Biodiversity Science and Ecological Engineering, Institute of Biodiversity Science, Fudan University, Shanghai, China; 2 Key Laboratory of Wetland Services and Restoration, Institute of Wetland Research, Chinese Academy of Forestry, Beijing, China; 3 School of Soil and Water Conservation, Beijing Forestry University, Beijing, China; Dauphin Island Sea Lab, United States of America

## Abstract

Soil disturbance has been widely recognized as an important factor influencing the structure and dynamics of plant communities. Although soil reworkers were shown to increase habitat complexity and raise the risk of plant invasion, their role in regulating the interactions between native and invasive species remains unclear. We proposed that crab activities, via improving soil nitrogen availability, may indirectly affect the interactions between invasive *Spartina alterniflora* and native *Phragmites australis* and *Scirpus mariqueter* in salt marsh ecosystems. We conducted a two-year mesocosm experiment consisting of five species combinations, i.e., monocultures of three species and pair-wise mixtures of invasive and native species, with crabs being either present or absent for each combination. We found that crabs could mitigate soil nitrogen depletion in the mesocosm over the two years. Plant performance of all species, at both the ramet-level (height and biomass per ramet) and plot-level (density, total above- and belowground biomass), were promoted by crab activities. These plants responded to crab disturbance primarily by clonal propagation, as plot-level performance was more sensitive to crabs than ramet-level. Moreover, crab activities altered the competition between *Spartina* and native plants in favor of the former, since *Spartina* was more promoted than native plants by crab activities. Our results suggested that crab activities may increase the competition ability of *Spartina* over native *Phragmites* and *Scirpus* through alleviating soil nitrogen limitation.

## Introduction

Biological invasions are one of the most serious threats to biodiversity and ecosystem functioning [1]. The interactions between invasive and native species play a critical role in regulating the structure and dynamics of plant communities [2]. Some of the interactions are mediated by other ecosystem components (e.g., nutrient availability, herbivores) [3]. The concept of “indirect effects” in communities began to draw ecologists’ attention two decades ago [4], and was soon introduced into bioinvasion studies [5]. For example, an invasive grass *Agrostis capillaris* led to a population proliferation of the herbivorous slug *Deroceras reticulatum*, imposing a negative impact on the native fern *Botrychium australe* [6]. Another example is that AM fungi strongly enhance the invader *Centaurea maculosa* to outcompete native *Festuca idahoensis* [7]. Indirect effects are implicit in several ecological theories (e.g., exploitative competition, apparent competition, indirect mutualism or facilitation, cascading effects) [4], most of which focus on the relationships between predator and prey (herbivore-primary producer) [3], [8], [9], host and parasite [10], or pollinator and plant [11], [12]. Crooks [13] reviewed the ecosystem-level consequences of abiotic resource modification (or “ecosystem engineering”) by invasive species. However, the role of native soil modifiers in mediating plant interactions remains largely unknown, although a recent study reported that grazers might mediate competition between two graminoid species via altering soil nutrient status [14].

Some organisms termed “ecosystem engineer” can increase habitat complexity by altering hydrological and edaphic conditions. Ecosystem engineers influence the growth and dispersal of both native and invasive plants, sometimes facilitating successful invasions [13]. As an example, the dam building beaver (*Castor canadensis*) could increase habitat heterogeneity, and thus increases the richness of herbaceous plants in the riparian zone in central Adirondacks, NY, USA [15]. In another case, the native cushion plant *Azorella monantha* could ameliorate harsh high-elevation conditions (e.g., low temperatures) and facilitate colonization by non-native species [16]. However, there is a lack of evidence for soil modifiers to mediate interactions between native and invasive species. We propose that native soil fauna play an important role in mediating the interactions between native and invasive plants by reworking the soil and altering nitrogen availability. Crabs are common burrowing species, and their activities can change the physical conditions such as soil moisture, temperature and aeration, which may in turn influence the microbial activities such as mineralization, nitrification and denitrification [17]. Furthermore, the nutrient-rich microhabitats created by crabs may promote invasion by non-native plants, as predicted by Davis’s fluctuating resource hypothesis [18]. Invasive species are most likely to invade post-disturbance habitats, as they often benefit more from increased resource availability due to faster growth and higher resource use efficiency.

We explored how the interactions between native and invasive plants could be mediated by disturbance from soil animals in a salt marsh. A mesocosm was established to mimic salt marsh conditions in the Yangtze River estuary, where *Phragmites australis* (“*Phragmites*” hereafter) and *Scirpus mariqueter* (“*Scirpus*” hereafter) are native, and *Spartina alterniflora* (“*Spartina*” hereafter) is exotic. Crabs are the most abundant native fauna dwelling in the estuary. The crab community consists mainly of *Helice tientsinensis* (“*Helice*” hereafter), *Chiromantes dehaani*, *Parasesarma plicatum* and *Uca arcuata*, with the average density being approximately 20 ind. m^−2^ [19]. Borrowing and soil transporting by crabs accelerate the turnover of sediments from deep layers to the surface, thereby playing an important role in regulating soil conditions [20]. The study of the indirect effect of native crab activities on the interaction between invasive and native plants may provide a novel way to explore the mechanisms of plant invasion. We addressed three specific questions: (i) Do native crabs affect soil nutrient availability? (ii) Does crab disturbance to the soil influence the performance of invasive *Spartina* and native *Scirpus* and *Phragmites*? (iii) Is *Spartina* more competitive than the two natives in response to crab disturbance?

## Materials and Methods

### Ethics Statement

All necessary permits were obtained for the described field studies. We are authorized by the Administrative Office of Shanghai Chongming Dongtan National Nature Reserve to carry out research activities including plant and crab collection in the entire area of the reserve. In addition, the crab *Helice* was an abundant species in the reserve, and was permitted to be appropriately fished for economic income.

### Study Species

All the plants and crabs used in this study were collected from the salt marsh at Chongming Dongtan in Yangtze River estuary (121° 50′ to 122° 05′ E, 31° 25′ to 31° 38′ N). *Spartina*, *Phragmites* and *Scirpus* are perennial clonal plants, expanding in salt marshes through both seed dispersal and vegetative propagation. Native *Phragmites* and *Scirpus* formed distinct zonation in Chongming Dongtan before the *Spartina* invasion. *Phragmites* occupied the high tidal zone and *Scirpus* dominated the low and intertidal zones. *Spartina* has been invading the Yangtze estuary since 1990s, and rapidly colonized tidal mudflats and salt marshes, driving a new pattern of zonation. The invasion success of *Spartina* may be attributable to its stronger tolerance to salinity than the natives, as well as greater leaf area index, higher maximal net photosynthetic rate, longer growing season, and higher use efficiency of light, water, and nitrogen [21], [22].

The crab *Helice* is one of the most abundant crab species at Chongming Dongtan, feeding on fresh plant, fauna and detritus [19]. The dietary composition of *Helice* is flexible, depending on food availability. The carapace width of adult *Helice* is ca. 20∼25 mm [23]. The burrow depth of *Helice* at Chongming Dongtan depends on the belowground structures of plant tissues. The deepest burrows occur in mudflat with an average depth of 38 cm, while the shallowest occur in *Spartina* habitats with an average depth of 15 cm [23]. In this study, *Helice* was selected as the source of disturbance in the mesocosm because it dwells in all types of habitats created by the three plants at Chongming Dongtan.

### Experimental Design

The experiment was conducted over two growing seasons (from May 2009 to November 2010) in a mesocosm at Fudan University in Shanghai (31°18′07′′ N, 121°29′47′′ E, China), about 10 km from the Yangtze River estuary. Forty cement plots (1×1 m^2^) were randomly assigned to ten treatments, i.e., five species combinations × two crab levels (absence vs. presence), with four replicates for each treatment. The plant species combinations included the monocultures of *Spartina*, *Phragmites* and *Scirpus*, and *Spartina*-*Phragmites* and *Spartina*-*Scirpus* mixtures. At the experimental setup, we transplanted 16 *Spartina* or *Phragmites* ramets per plot in their monocultures, and alternately arranged 8 ramets of each species in mixture plots. In comparison, we transplanted 32 *Scirpus* ramets per plot in its monocultures and 16 *Scirpus* ramets in mixtures with 8 *Spartina* ramets because *Scirpus* is much smaller than the other two species. The crab-present treatment was established by placing 20 adult *Helice* individuals (with a mean carapace width of 22 mm and a sex ratio of 1 : 1) captured from Chongming Dongtan into each plot. The plots were filled with a mixture of clay (60%) and sand (40%) to a depth of 30 cm. Soil salinity in all plots was adjusted to 15, which is typical of Chongming Dongtan, by irrigating the plots with NaCl solution. In May 2009, we excavated the ramets of three species with rhizomes from Chongming Dongtan and acclimatized them in fresh water for two days prior to transplanting. The crabs were added in June 2009 after all the ramets had established. The upper edge of each plot was encompassed by slippery plastic sheets of 15 cm in width to prevent crab escaping. The few died crabs found were replaced with similar-sized healthy individuals of the same sex to maintain the disturbance intensity in crab-present plots.

### Measurements of Plant Performance and Soil Nitrogen

We here focused on the interactions between *Spartina* and the two native species, so harvesting plants asynchronously may lead to a bias in estimating the species interactions. Therefore, we harvested all aboveground biomass in late October in both years, when *Spartina* had matured whereas *Scirpus* had withered and *Phragmites* was still flowering. Once the aboveground plant was reaped, the number of ramets and total fresh biomass were recorded for each plot. We randomly chose ten ramets per species from each plot to obtain the mean ramet height and fresh-mass. The ten ramets were oven-dried at 60°C to constant weight and weighed to determine mean ramet dry-mass. The total aboveground dry-mass of each plot was calculated by dividing the fresh-mass by the ratio of fresh to dry-mass of the ten collected ramets.

Soil samples were taken in May 2009 and October 2010. Five soil cores (3.4 cm in diameter) were sampled to a depth of 25 cm from each plot and mixed. Subsamples of 100 g of mixed soil from each plot were collected and sealed in ziplock bags for subsequent determination of soil properties. In October 2010, the belowground plant tissues were flushed from the remaining soil samples with running water and oven-dried at 60°C to constant weight. As it was difficult to discriminate the three species based on belowground traits, we could only estimate the plot-level belowground biomass, which was calculated by dividing the oven-dried biomass by the ratio of sampled area (i.e., the total intersection area of five soil cores) to plot area (1 m^2^). The soil samples for determining chemical properties were air dried and ground to pass through a 100-mesh sieve. Fine roots were then manually removed. We used soil ammonium (NH_4_^+^-N) and nitrate (NO_3_^–^N) concentrations as indicators of nitrogen availability. Ten grams of sieved soil was extracted with 40 ml of 2 M KCl solution. The extracts were then stirred for 1 h at 30°C and filtered through medium-speed qualitative filter paper. The NH_4_^+^-N and NO_3_^–^N concentrations were determined for the filtrates with a discrete chemistry analyzer (SMARTCHEM™200, Westco Scientific Instruments, Inc., USA). Soil total nitrogen (TN) was determined for the sieved soil with a N/C analyzer (FlashEA1112 Series, Thermo Inc., Milan, Italy).

We measured the number and total volume of crab burrows in each plot to assess the disturbance intensity in 2010 after plants had been reaped. The volume of burrows was calculated from their diameters and depths under the assumption that the burrows were approximately cylindrical.

### Data Analysis

We carried out all analyses using software R version 3.0.1 [24]. The relative changes of soil NH_4_^+^-N, NO_3_^−^ -N and TN over the two years were calculated by dividing the concentration in October 2010 (final) by the value in May 2009 (initial). We used multivariate analysis of variance (MANOVA) to examine the variation in soil nitrogen availability among treatments at the initial state, with the overall concentrations of NH_4_^+^-N, NO_3_^−^ -N and TN being dependent variables. The five species combinations were then divided into two groups to compare the effects of crabs and species combinations on soil nitrogen. The first group (“*Spartina*-present plots” hereafter) consisted of *Spartina* monocultures and *Spartina*-*Phragmites/Scirpus* mixtures, and the second group (“*Spartina*-absent plots” hereafter) consisted of *Phragmites* and *Scirpus* monocultures. The effects of crabs and species combinations on the overall relative changes of three soil nitrogen forms were tested using MANOVA for the two groups, respectively. Subsequently, two-way analyses of variance (ANOVA) were conducted for each of the relative changes in NH_4_^+^-N, NO_3_^−^ -N and TN for the two groups, respectively. The differences in density and total volume of crab burrows among the five species combinations in crab-present plots were tested using one-way ANOVA.

A linear mixed-effects model, within the “nlme” package, was used to test the effects of crab, species and plot types on the overall plant performance. The response variables were mean height and biomass of ramets, density and total aboveground biomass. The model included “crab” (present or absent), “species” (*Spartina*, *Phragmites* or *Scirpus*), “plot type” (mixture or monoculture) and their interactions as fixed factors, and “year” as a random factor. For each species, mixed model ANOVAs were applied to each of the four plant traits, with “crab”, “species combination” and their interaction as fixed factors and “year” as a random factor. In addition, the effects of crabs and five species combinations on the total belowground biomass in 2010 were tested using two-way ANOVA. The ratios of plant performance under crab disturbance to those without crabs (*R_crab_*) were calculated to facilitate comparison of crab effects among species combinations.

We used two mixed model ANOVAs to further test whether crab activities regulate the interactions between native and the two invasive species. Only data from mixture plots were used for this purpose, the model included “crab”, “species” (*Spartina* vs. *Phragmites* in the first ANOVA and *Spartina* vs. *Scirpus* in the second ANOVA) and their interaction as fixed terms and “year” as a random factor. A significant “crab × species” term indicates a change in interspecific relationship attributable to crabs. This analysis was applied to each of the four plant traits. The response variables were log-transformed to meet the assumptions of normality and homoscedasticity in all above analyses.

## Results

### Soil Nitrogen

**Figure 1:**
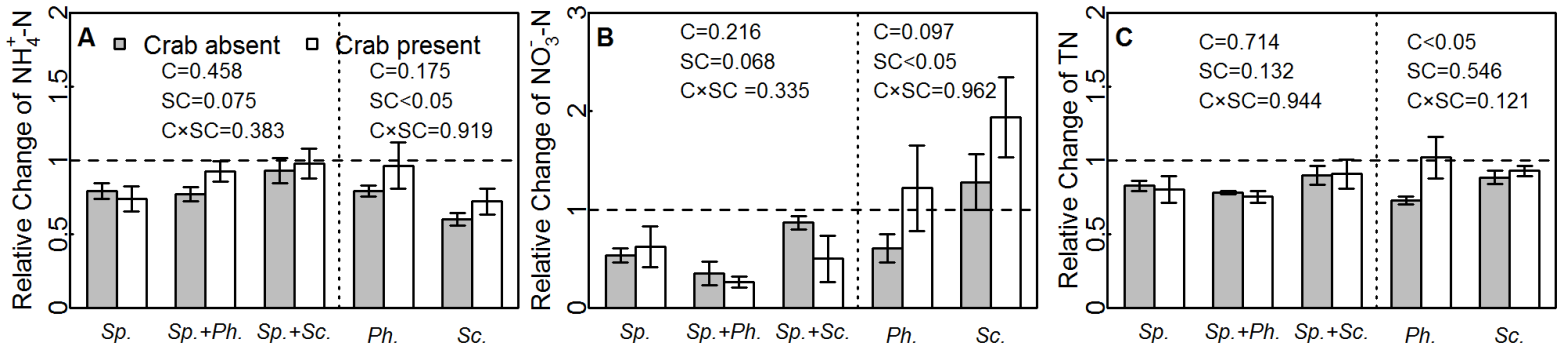
The relative change of soil nitrogen. The effects of crab and species combinations on the relative changes of NH_4_^+^-N (mean ± SE, A), NO_3_^−^ -N (mean ± SE, B), and TN (mean ± SE, C) were tested using two-way ANOVA. The *P*-values of the effects of crabs and species combinations on relative change of nitrogen are given, where “C” stands for crab treatment and “SC” for species combination. The vertical dash line divides the species combinations into *Spartina*-present plots and *Spartina*-absent plots, and the horizontal dash line at 1 distinguishes nitrogen increase from decrease.

Initial soil nitrogen availability did not differ among treatments (MANOVA: crab, *Pillai’s trace _1,30_* = 0.039, *F_3,28_* = 0.38, *P* = 0.772; species combination, *Pillai’s trace_4,30_* = 0.51, *F_12,90_* = 1.53, *P* = 0.128; crab × species combination: *Pillai’s trace_4,30_* = 0.19, *F_3,12_* = 0.49, *P = *0.915). Soil NH_4_^+^-N, NO_3_^−^ -N and TN decreased at the end of the experiment, as indicated by <1 relative changes (Figure 1), except for the NO_3_^−^ -N in native monocultures (Figure 1B). Final soil nitrogen was significantly affected by crabs in *Spatina*-absent plots (*Pillai’s trace_1,12_* = 0.53, *F_3,10_* = 3.73, *P*<0.05), but not in *Spartina-*present plots (*Pillai’s trace_1,18_* = 0.15, *F_3,16_* = 0.97, *P* = 0.432). The crabs appeared to alleviate nitrogen limitation in *Spartina*-absent plots, as indicated by higher relative changes in nitrogen (see *Ph.* and *Sc.* in Figure 1A–C), although a significant crab effect was only found for TN (Figure 1C, Table S1). The species combination affected the soil nitrogen in both *Spartina*-present (*Pillai’s trace_1,18_* = 0.59, *F_6,34_* = 2.37, *P*<0.05) and *Spartina*-absent plots (*Pillai’s trace_1,12_* = 0.57, *F_3,10_* = 4.37, *P*<0.05), as NH_4_^+^-N and NO_3_^−^ -N (but not TN) varied among species combinations (Figure 1). The density and volume of crab burrows did not differ among species combinations (density: *F_4,15_* = 1.59, *P = *0.229; total volume: *F_4,15_* = 1.33, *P = *0.303).

### Plant Performance

**Figure 2:**
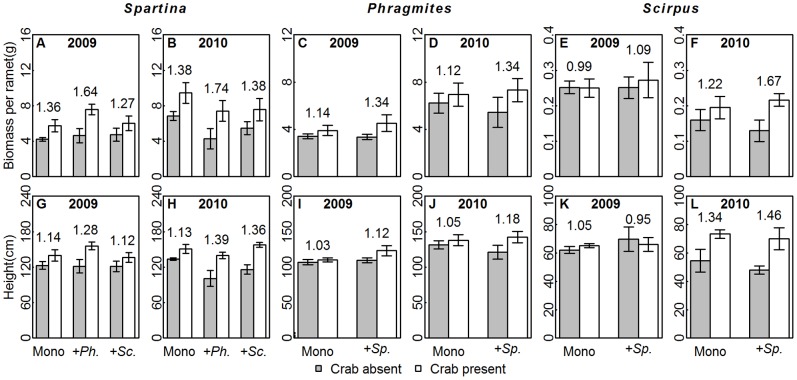
The plant performance at ramet-level. The biomass per ramet (mean±SE, A–F) and height (mean ± SE, G–L) of three species with/without crab disturbance in five species combinations are presented. The categories on X axis present different species combinations. “Mono”, “+*Sp.*”, “+*Ph.*” and “+*Sc.*” are abbreviations for “monocultures”, “with *Spartina*”, “with *Phragmites*” and “with *Scirpus*”, respectively. The *R_crab_*s of three species in specific species combinations were given above the bars.

**Figure 3:**
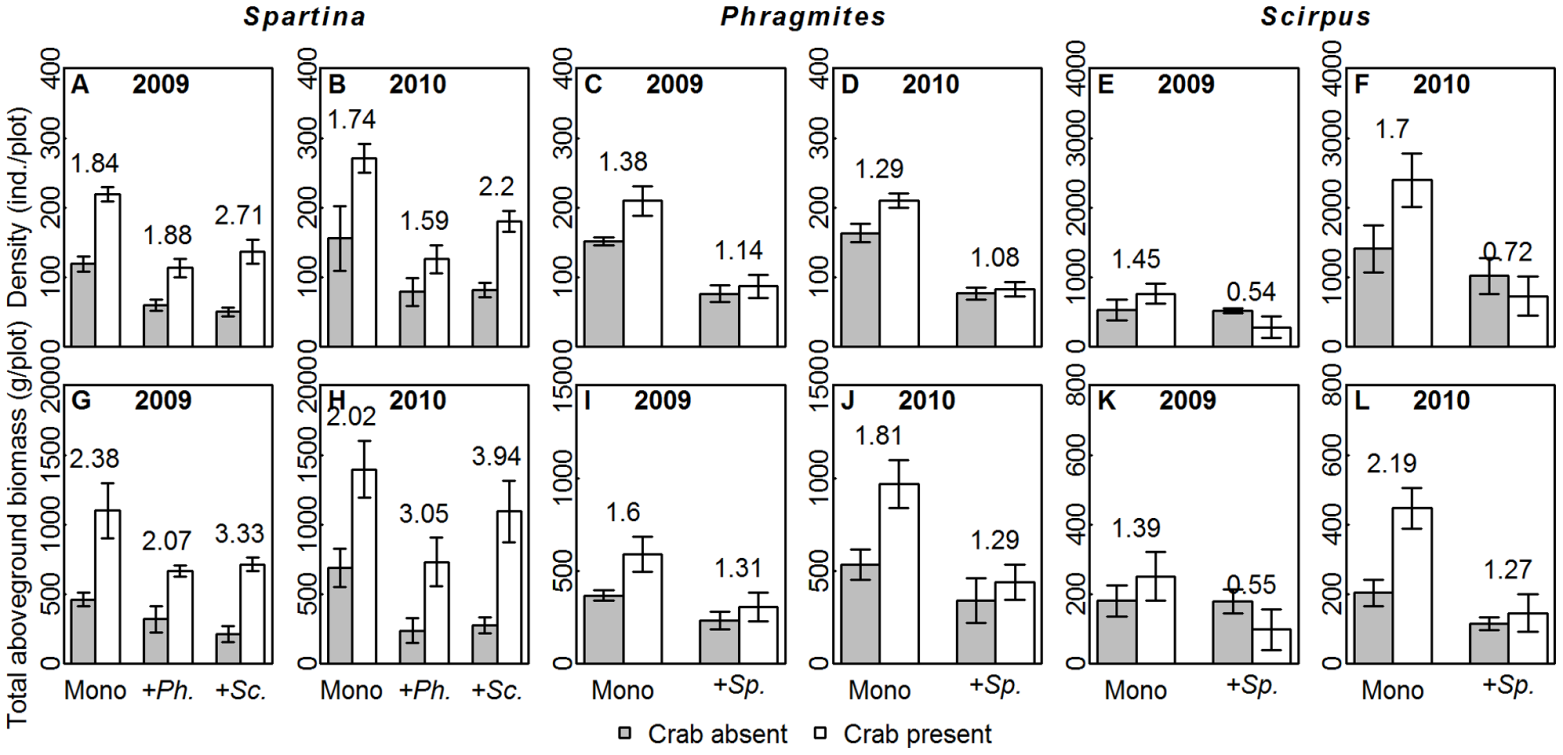
The plant performance at plot-level. The density (mean ± SE, A–F) and total aboveground biomass (mean±SE, G–L) per plot of three species with/without crab disturbance in five species combinations are presented. The categories on X axis present different species combinations. “Mono”, “+*Sp.*”, “+*Ph.*” and “+*Sc.*” are abbreviations for “monocultures”, “with *Spartina*”, “with *Phragmites*” and “with *Scirpus*”, respectively. The *R_crab_*s of three species in specific species combinations are given above the bars.

**Figure 4:**
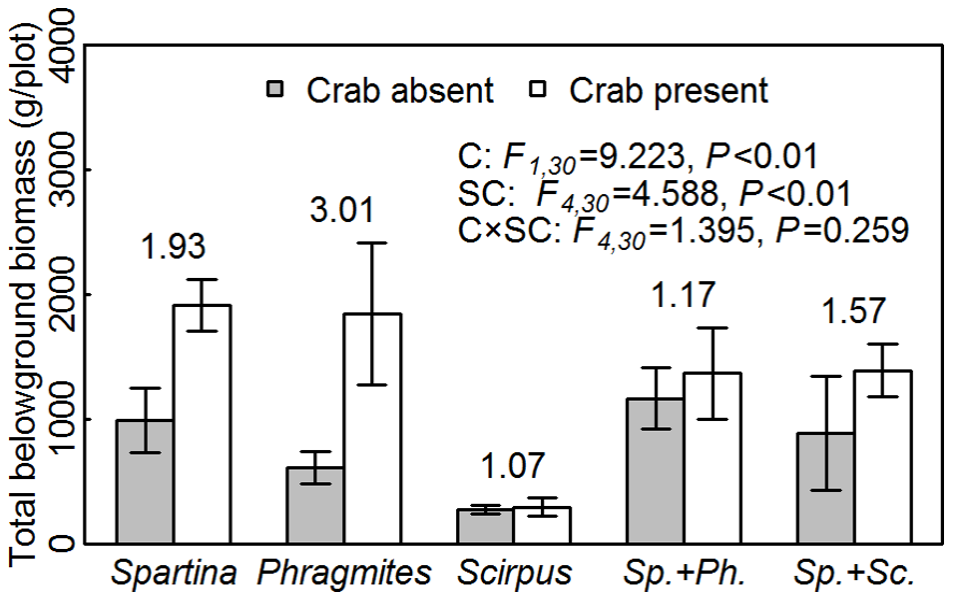
The total belowground biomass (mean ± SE) with/without crab disturbance in five species combinations. The categories on X axis present different species combinations. “*Sp.*”, “*Ph.*” and “*Sc.*” are abbreviations for “*Spartina*”, “*Phragmites”* and “*Scirpus*”, respectively. The *F*-values and *P*-values of the effects of crabs and five species combinations on belowground yield are given, where “C” stands for crab treatments and “SC” stands for species combinations. The *R_crab_*s of three species in specific species combinations are given above the bars.

Plant performance varied with crab disturbance, species and plot types, as well as their interactions (Table 1, Table S2). All the three species in crab-present plots outperformed that in crab-absent plots (Figure 2, 3, Table S3), except for the biomass per ramet and density of *Scirpus*. In addition, the total belowground biomass of all five species combinations increased in response to crab disturbance (Figure 4). The three species, however, were promoted to different degrees, as shown by the significant “crab × species” term (Table 1). The *R_crab_*s of *Spartina* were generally higher than those of native species (Figure 2, 3), suggesting that *Spartina* benefited more from crab activities than native species. Furthermore, the ramet-level performance (i.e., biomass per ramet and height) of both the native and invasive plants were not affected by species combination and the interaction with crabs (Table S3), compared with the significant “crab × species combination” terms for the plot-level performance (i.e., density, Table S3). Meanwhile, the *R_crab_*s of plot-level performance of three species in monocultures were generally higher than that of ramet-level performance, e.g. the *R_crab_*s of total aboveground biomass of *Spartina* in monocultures (Figure 3G) were higher than that of biomass per ramet (Figure 2G). This suggests that the number, but not size, of ramets was more sensitive to crabs in different species combinations.

**Table 1:** Summary for ANOVA of the linear mixed-effects model^a^ for effects of species, plot types and crabs on overall plant performance^b^.

Source of variance	*numDf*	*denDf*	*F*	*P*	
Species	2	99	57.24	<0.001	***^c^
Plot type	1	99	56.30	<0.001	***
Crab	1	99	50.06	<0.001	***
Species×Plot type	2	99	0.07	0.932	
Species×Crab	2	99	14.19	<0.001	***
Plot type×Crab	1	99	2.33	0.130	
Species× plot type ×crab	2	99	3.24	0.043	*

a More information of the linear mix model is supplied in Table S2.

b “plant performance” was combined of biomass per ramet, height, density and total aboveground biomass.

c Asterisks indicate level of significance (* <0.05, ** <0.01, *** <0.001).

### Interactions between Invasive and Native Plants

**Figure 5:**
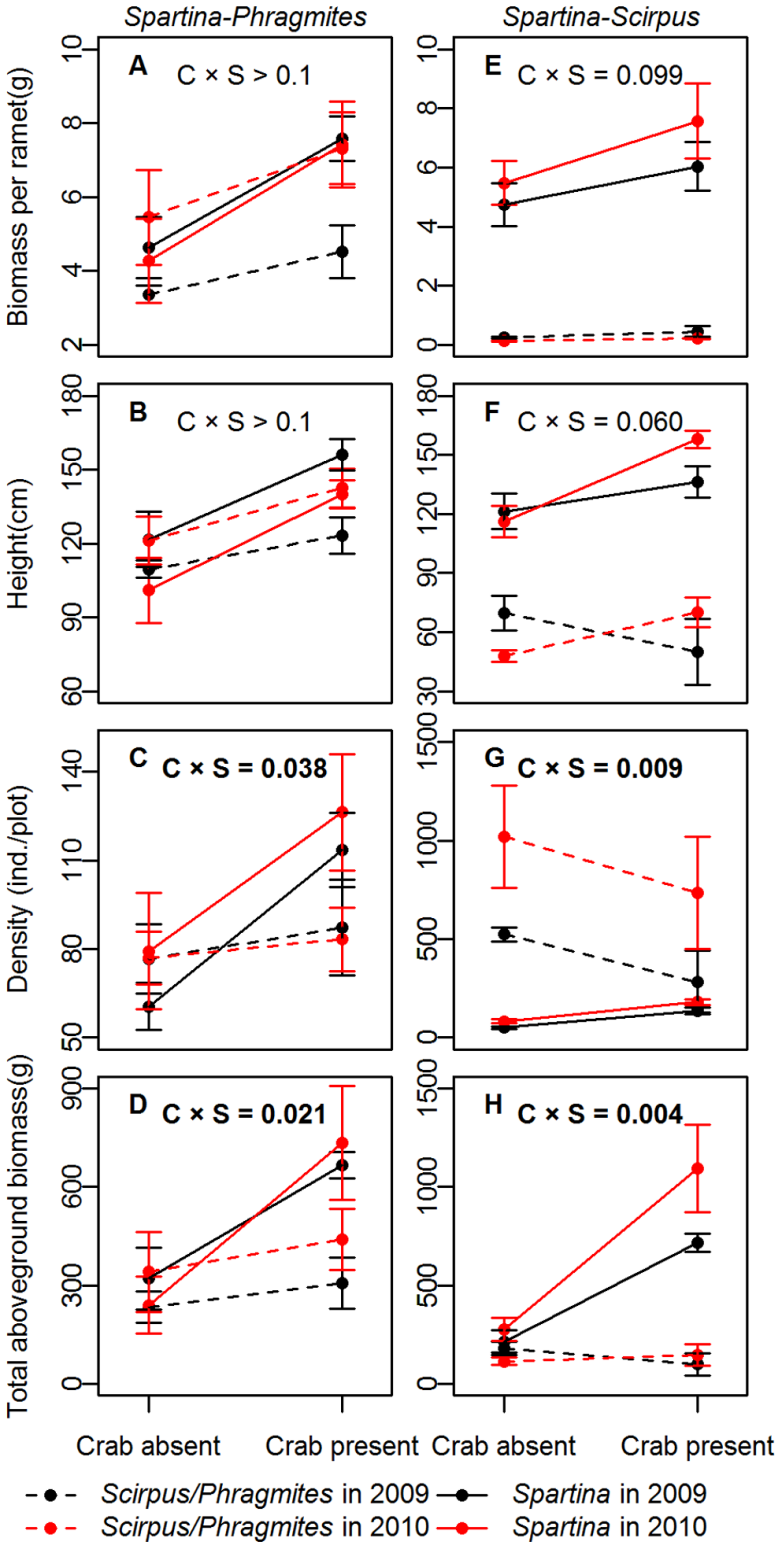
The performance of invasive vs. native plant in mixtures with or without crab disturbance. The biomass per ramet (mean ± SE, A and E), height (mean ± SE, B and F), density (mean ± SE, C and G) and aboveground biomass (mean ± SE, D and H) of invasive and native species in mixtures are presented. The *P*-values of interactive effects of “crab × species” are given, where “C” stands for crab treatments and “S” stands for species.

In mixtures, *Spartina* was facilitated more by crabs than natives (Figure 5), as demonstrated by significant “crab × species” terms for density and total aboveground biomass (Figure 5C, D, G, H, Table S4). Ramet-level performance, however, showed similar responses to crab disturbance between invasive and native plants (Figure 5A, B, E, F).

## Discussion

Soil animals regulate plant communities through herbivory and soil reworking (i.e., ecosystem engineering effects). Our results suggested that crabs mediated the interactions between native and invasive plants through soil reworking. Herbivory by crabs was observed to be negligible (see monocultures in Figure 2, 3), although *Helice* might partially feeds on these plants [19]. Therefore, herbivory was unlikely to alter the relationship between invasive and native plants in this study.

As one of the important drivers of plant community structure, soil nutrient availability may determine the relative dominance of species [25]. Low nitrogen availability, which often occurs in salt marsh ecosystems [26], [27], can suppress growth and competitive ability of potentially dominant species [28], [29]. Nitrogen limitation may occur in the mesocosm, as the total inorganic nitrogen (sum of NH_4_^+^-N and NO_3_^−^ -N) in the mesocosm ranged from 5 to 8 µg g^−1^ and TN ranged from 0.31 to 0.67 mg g^−1^, falling in the range observed in Chongming Dongtan salt marsh (total inorganic nitrogen from 4 to 15 µg g^−1^ [30] and TN from 0.2 to 0.7 mg g^−1^ [31]). The decreases in nitrogen (Figure 1) might result from greater plant uptake than nitrogen supply by decomposition. However, mineralization could be accelerated by enhanced microbial activities, which are regulated by the physical and chemical environments. Crab activities, such as burrowing, litter fragmentation, feeding and excretion, can create better conditions for decomposition [17]. The crab effects on nitrogen were found in *Spartina*-absent plots, where the relative changes in nitrogen was higher than in crab-present plots (Figure 1B, C). The lack of crab effects on nitrogen in *Spartina*-present plots might be the result of higher nitrogen consumption by fast-growing *Spartina*, which might have offset the improved nitrogen availability by crabs. Previous studies have demonstrated that increased nitrogen storage in plant tissues may neutralize the changes in the soil nitrogen caused by fertilization, especially in nitrogen-limiting ecosystems [32]–[34].

The plant performance, both of aboveground and belowground parts (Figures 2–4), had been improved due to the alleviated soil conditions. Among the three species, alien *Spatina* responded most sensitively to crab disturbance, as the *R_crab_*s of *Spartina* in monocultures were generally higher than the other two species (Figure 2, 3). The higher benefits that the invasive *Spartina* (C_4_ photosynthetic pathway) received from crab disturbance could be attributed to the CO_2_-concentrating mechanism of C_4_ plants, in which each unit of nitrogen has a higher CO_2_ fixation efficiency than C_3_ species [35], [36]. Therefore, even slight increases in nitrogen availability may lead to marked enhancement in *Spartina* performance. Nitrogen limitation is proposed to prevent invasion, and nitrogen addition could increase the growth of invasive plants in salt marsh and desert [25], [37]. It follows that the crab activities could promote *Spartina* invasion in saltmarsh through improving nitrogen availability, as predicted by Davis [18], that the availability of limiting nitrogen will increase the vulnerability of a community to invasion.

The interactions between invasive and native species were altered by crab disturbance. In mixtures, *Spartina* benefited more from crab activities than natives, potentially leading to a competitive superiority of *Spartina* under improved nitrogen conditions (Figure 5). The enhanced competitive ability of *Spartina* in crab-present mixtures could be explained by its higher growth rate, CO_2_ fixation efficiency and nitrogen consumption rate. Wang [38] also found that nitrogen addition favored invasive *Spartina* over native *Phragmites* under high-salinity conditions, while crabs played a role of improving nitrogen availability in this study, as discussed above. Therefore, we conclude that native soil reworkers (or ecosystem engineers such as crabs) are capable of altering the interactions among plant species through alleviating resource limitations.

We found that the crabs promoted the plants more effectively at the plot-level based on the significant “crab × species combinations” effects on density for all three species (Table 1), with the *R_crab_*s of total aboveground biomass (Figure 3G–L) being generally higher than that of biomass per ramet (Figure 3A–F). In other words, the plants responded to crabs’ modification on soils primarily through increased number, rather than size, of ramets. It was similar to Tyler’s study [39], that nitrogen input could promote the spread of invasive *Spartina* sp. into previously unvegetated mudflats in estuaries of the western United States, and high nitrogen loading areas were characterized by higher total aboveground biomass and ramet density.

It should be noted that *Spartina* may use different strategies to compete with *Phragmites* and *Scirpus*. At the ramet-level, the *R_crab_*s of *Spartina* in *Spartina*-*Phragmites* mixtures were higher than those in *Spartina*-*Scirpus* mixtures (Figure 2A, B, G, H), while the pattern reversed at the plot-level (Figure 3A, B, G, H). This implies that *Spartina* tended to invest more in vegetative growth to compete with *Phragmites*, whereas invested more in the clonal expansion when competing with *Scirpus*. This speculation could also be supported by the change of belowground biomass. The increase of belowground biomass in *Spartina*-*Phragmites* mixtures was not as distinct as that in their monocultures (Figure 4), implying that crabs might influence them more at aboveground in mixtures. Meanwhile the increase of belowground biomass in *Spartina*-*Scirpus* mixtures under crab disturbance might primary come from the *Spartina*, since the *R_crab_*s were *Spartina* mococultures>*Spartina*- *Scirpus* mixtures>*Scirpus* monocultures (Figure 4).The different strategies that *Spartina* used might associated with the phylogenetic relatedness among competitors, i.e., both *Spartina* and *Phragmites* belong to Gramineae whereas *Scirpus* is a member of Cyperaceae. Species of close phylogenetic relatedness tend to have similar phenotype and niche, and thus compete more intensely [40]. *Spartina* ramets need to grow higher and larger to compete for space and light with similar sized *Phragmites* ramets. In contrast, the size superiority of *Spartina* over *Scirpus* might allow *Spartina* to invest more in belowground expansion and clonal growth.

This study suggests that native crabs may accelerate the invasion of *Spartina* through alleviating nitrogen limitation, which helps understand and predict the invasion success in natural salt marshes. However, other environmental factors should not be overlooked when extrapolating this conclusion from the mesocosm to natural ecosystems. In the Chongming Dongtan salt marsh, the role of crabs may rely on their habitat preferences. For example, crabs prefer to dwell in *Spartina* communities [41] and form a mutualistic relationship between crabs and *Spartina*, thereby promoting *Spartina* invasion. In addition, crab herbivory should not be neglected in natural ecosystems [42]. In conclusion, we suggest that native crab disturbance to soil can mediate the interspecific interactions between invasive *Spartina* and native *Phragmites*/*Scirpus* through modifying soil nitrogen availability. Although crab activities may facilitate *Spartina* invasion in salt marshes, the results obtained from the mesocosm system need to be further tested in the field.

## Supporting Information

Table S1 **The ANOVA table of crab treatments and plant combinations on three nitrogen forms either in Spartina-present or -absent plots.**
(DOCX)

Table S2 **The linear mixed-effects model for effects of species, plot type and crab, with year as random factors, on the overall plant performance.**
(DOCX)

Table S3 **The mixed ANOVA model for the effects of crab, plant combinations and their interactions on the biomass per ramet, height, density and total aboveground biomass of each species, with year as the random factor.**
(DOCX)

Table S4 **The mixed ANOVA model for the effects of crab, species and their interactions on the biomass per ramet, height, density and total aboveground biomass of each species in mixtures, with year as the random factor.**
(DOCX)
