# Supplementary material for: Crabs Mediate Interactions between Native and Invasive Salt Marsh Plants: A Mesocosm Study
Source: PLoS One. 2013 Sep 4;8(9):e74095. doi: 10.1371/journal.pone.0074095 (PMC3762776; doi:10.1371/journal.pone.0074095)
Supplement: Table S1 — The ANOVA table of crab treatments and plant combinations on three nitrogen forms either in Spartina-present or -absent plots. (DOCX) [file pone.0074095.s001.docx]

**Table S1. The ANOVA table of crab treatments and plant combinations on three nitrogen forms either in *Spartina*-present or -absent plots.**

|  |  | *Spartina*-present plots | | | |  | *Spartina*-absent plots | | | | |
| --- | --- | --- | --- | --- | --- | --- | --- | --- | --- | --- | --- |
|  | Source of variance | *df* | *MS* | *F* | *P* |  | *df* | *MS* | *F* | *P* |  |
| NH_4_^+^-N | Crab | 1 | 0.01608 | 0.704 | 0.412 |  | 1 | 0.10154 | 1.723 | 0.213 |  |
|  | Species combination | 2 | 0.07089 | 3.105 | 0.069 | + | 1 | 0.29587 | 5.02 | 0.049 | * |
|  | Crab × Species combination | 2 | 0.02171 | 0.951 | 0.405 |  | 1 | 0.00023 | 0.004 | 0.952 |  |
|  | Residual | 18 | 0.02283 |  |  |  | 12 | 0.05894 |  |  |  |
| NO_3_^-^ -N | Crab | 1 | 0.08914 | 1.07 | 0.314 |  | 1 | 0.8944 | 2.534 | 0.137 |  |
|  | Species combination | 2 | 0.30423 | 3.654 | 0.047 | * | 1 | 2.0333 | 5.76 | 0.034 | * |
|  | Crab × Species combination | 2 | 0.10545 | 1.266 | 0.306 |  | 1 | 0.0072 | 0.02 | 0.889 |  |
|  | Residual | 18 | 0.08327 |  |  |  | 12 | 0.353 |  |  |  |
| TN | Crab | 1 | 0.00068 | 0.007 | 0.935 |  | 1 | 0.13141 | 5.914 | 0.032 | * |
|  | Species combination | 2 | 0.23173 | 2.371 | 0.122 |  | 1 | 0.01529 | 0.688 | 0.423 |  |
|  | Crab × Species combination | 2 | 0.00614 | 0.063 | 0.939 |  | 1 | 0.06841 | 3.079 | 0.104 |  |
|  | Residual | 18 | 0.09772 |  |  |  | 12 | 0.02222 |  |  |  |

Asterisks indicate level of significance (＋ <0.1, * <0.05, ** <0.01, *** <0.001).
