# Supplementary material for: Crabs Mediate Interactions between Native and Invasive Salt Marsh Plants: A Mesocosm Study
Source: PLoS One. 2013 Sep 4;8(9):e74095. doi: 10.1371/journal.pone.0074095 (PMC3762776; doi:10.1371/journal.pone.0074095)
Supplement: Table S2 — The linear mixed-effects model for effects of species, plot type and crab, with year as random factors, on the overall plant performance. (DOCX) [file pone.0074095.s002.docx]

**Table S2. The linear mixed-effects model for effects of species, plot type and crab, with year as the random factor, on the overall plant performance.**

|  | Coeffecient | Std.Error | *df* | *t* | *P* |
| --- | --- | --- | --- | --- | --- |
| (Intercept) | -0.515 | 0.201 | 99 | -2.560 | 0.012 |
| speciesph | 0.087 | 0.238 | 99 | 0.365 | 0.716 |
| speciessc | -0.472 | 0.238 | 99 | -1.979 | 0.051 |
| plottypemono | 0.976 | 0.238 | 99 | 4.092 | <0.001 |
| craby | 1.430 | 0.195 | 99 | 7.345 | <0.001 |
| speciesph:plottypemono | -0.380 | 0.364 | 99 | -1.042 | 0.300 |
| speciessc:plottypemono | -0.748 | 0.364 | 99 | -2.054 | 0.043 |
| speciesph:craby | -1.108 | 0.337 | 99 | -3.286 | 0.001 |
| speciessc:craby | -1.906 | 0.337 | 99 | -5.652 | <0.001 |
| plottypemono:craby | -0.259 | 0.337 | 99 | -0.768 | 0.444 |
| speciesph:plottypemono:craby | 0.640 | 0.515 | 99 | 1.244 | 0.217 |
| speciessc:plottypemono:craby | 1.309 | 0.515 | 99 | 2.541 | 0.013 |

Note: this model was fitted by Restricted Maximum Likelihood (REML) method, with the Akaike information criterion (AIC) as 220.97, Bayesian information criterion (BIC) as 257.44, and Log-Likelihood as -96.48. The Standard deviation (StdDev) of residual for the random factor ‘year’ was 0.5505, with the StdDev of the intercept as 0.2075.
