# Supplementary material for: Crabs Mediate Interactions between Native and Invasive Salt Marsh Plants: A Mesocosm Study
Source: PLoS One. 2013 Sep 4;8(9):e74095. doi: 10.1371/journal.pone.0074095 (PMC3762776; doi:10.1371/journal.pone.0074095)
Supplement: Table S3 — The mixed ANOVA model for the effects of crab, plant combinations and their interactions on the biomass per ramet, height, density and total aboveground biomass of each species, with year as the random factor. (DOCX) [file pone.0074095.s003.docx]

**Table S3. The mixed ANOVA model for the effects of crab, plant combinations and their interactions on the biomass per ramet, height, density and total aboveground biomass of each species, with year as the random factor.**

|  |  | *Spartina* | | | | | *Phragmites* | | | | | *Scirpus* | | | | |
| --- | --- | --- | --- | --- | --- | --- | --- | --- | --- | --- | --- | --- | --- | --- | --- | --- |
|  | Source of variance | *df* | *MS* | *F* | *P* |  | *df* | *MS* | *F* | *P* |  | *df* | *MS* | *F* | *P* |  |
| Height | Year | 1 | 0.659 |  |  |  | 1 | 3447 |  |  |  | 1 | 0.176 |  |  |  |
|  | Crab | 1 | 8969 | 29.485 | <0.001 | *** | 1 | 991.5 | 6.201 | 0.019 | * | 1 | 288.8 | 0.965 | 0.335 |  |
|  | Species combination | 2 | 426 | 0.7 | 0.502 |  | 1 | 47.8 | 0.299 | 0.588 |  | 1 | 154.8 | 0.517 | 0.478 |  |
|  | Crab × Species combination | 2 | 795 | 1.306 | 0.282 |  | 1 | 318 | 0.299 | 0.169 |  | 1 | 192 | 0.642 | 0.43 |  |
|  | Residual | 41 | 12471 |  |  |  | 27 | 159.9 |  |  |  | 27 | 299 |  |  |  |
| Biomass per ramet | Year | 1 | 0.754 |  |  |  | 1 | 0.082 |  |  |  | 1 | 2.051 |  |  |  |
|  | Crab | 1 | 2.56 | 17.724 | <0.001 | *** | 1 | 0.019 | 5.621 | 0.025 | * | 1 | 0.701 | 5.227 | 0.03 | * |
|  | Species combination | 2 | 0.086 | 0.593 | 0.557 |  | 1 | 0.0003 | 0.095 | 0.76 |  | 1 | 0.037 | 0.275 | 0.604 |  |
|  | Crab × Species combination | 2 | 0.118 | 0.819 | 0.448 |  | 1 | 0.004 | 1.37 | 0.252 |  | 1 | 0.302 | 2.256 | 0.144 |  |
|  | Residual | 41 | 0.144 |  |  |  | 27 | 0.003 |  |  |  | 27 | 0.134 |  |  |  |
| Density | Year | 1 | 12805 |  |  |  | 1 | 32 |  |  |  | 1 | 1525 |  |  |  |
|  | Crab | 1 | 83333 | 61.887 | <0.001 | *** | 1 | 7320 | 11.747 | 0.002 | ** | 1 | 0.3 | 0.003 | 0.953 |  |
|  | Species combination | 2 | 42210 | 31.347 | <0.001 | *** | 1 | 84666 | 135.866 | <0.001 | *** | 1 | 915.7 | 12.338 | 0.002 | ** |
|  | Crab × Species combination | 2 | 3558 | 2.643 | 0.083 | + | 1 | 3828 | 6.143 | 0.019 | * | 1 | 550.1 | 7.412 | 0.011 | * |
|  | Residual | 41 | 1347 |  |  |  | 27 | 623 |  |  |  | 27 | 74.2 |  |  |  |
| Aboveground biomass | Year | 1 | 0.191 |  |  |  | 1 | 1.112 |  |  |  | 1 | 20336 |  |  |  |
|  | Crab | 1 | 13.948 | 56.644 | <0.001 | *** | 1 | 1.396 | 8.119 | 0.008 | ** | 1 | 34441 | 3.066 | 0.091 | + |
|  | Species combination | 2 | 2.542 | 10.324 | <0.001 | *** | 1 | 3.65 | 21.226 | <0.001 | *** | 1 | 146867 | 13.075 | 0.001 | ** |
|  | Crab × Species combination | 2 | 0.308 | 1.251 | 0.297 |  | 1 | 0.08 | 0.466 | 0.5 |  | 1 | 66204 | 5.894 | 0.022 | * |
|  | Residual | 41 | 0.246 |  |  |  | 27 | 0.172 |  |  |  | 27 | 11233 |  |  |  |

Asterisks indicate level of significance (＋ <0.1, * <0.05, ** <0.01, *** <0.001).
