# Supplementary material for: Crabs Mediate Interactions between Native and Invasive Salt Marsh Plants: A Mesocosm Study
Source: PLoS One. 2013 Sep 4;8(9):e74095. doi: 10.1371/journal.pone.0074095 (PMC3762776; doi:10.1371/journal.pone.0074095)
Supplement: Table S4 — The mixed ANOVA model for the effects of crab, species and their interactions on the biomass per ramet, height, density and total aboveground biomass of each species in mixtures, with year as the random factor. (DOCX) [file pone.0074095.s004.docx]

**Table S4. The mixed ANOVA model for the effects of crab, species and their interactions on the biomass per ramet, height, density and total aboveground biomass of each species in mixtures, with year as the random factor.**

|  |  | *Spartina-Phragmites* mixtures | | | | | *Spartina-Scirpus* mixtures | | | | |
| --- | --- | --- | --- | --- | --- | --- | --- | --- | --- | --- | --- |
|  |  | *df* | *MS* | *F* | *P* |  | *df* | *MS* | *F* | *P* |  |
| Height | Year | 1 | 16.53 |  |  |  | 1 | 112.5 |  |  |  |
|  | Crab | 1 | 5866 | 16.609 | <0.001 | *** | 1 | 1744 | 4.524 | 0.0427 | * |
|  | Species | 1 | 243 | 0.689 | 0.413 |  | 1 | 43181 | 112 | <0.001 | *** |
|  | Crab × Species | 1 | 744 | 2.105 | 0.158 |  | 1 | 1492 | 3.87 | 0.059 | + |
|  | Residual | 27 | 353 |  |  |  | 27 | 386 |  |  |  |
| Biomass per ramet | Year | 1 | 9.517 |  |  |  | 1 | 1.825 |  |  |  |
|  | Crab | 1 | 41.72 | 11.622 | 0.002 | ** | 1 | 6.79 | 4.107 | 0.052 | + |
|  | Species | 1 | 5.26 | 1.465 | 0.236 |  | 1 | 259.41 | 156.804 | <0.001 | *** |
|  | Crab × Species | 1 | 4.7 | 1.31 | 0.262 |  | 1 | 4.82 | 2.916 | 0.099 | + |
|  | Residual | 27 | 3.59 |  |  |  | 27 | 1.65 |  |  |  |
| Density | Year | 1 | 385 |  |  |  | 1 | 5.681 |  |  |  |
|  | Crab | 1 | 6815 | 9.264 | 0.005 | ** | 1 | 0.807 | 0.546 | 0.466 |  |
|  | Species | 1 | 1526 | 2.075 | 0.161 |  | 1 | 11.269 | 7.621 | 0.01 | * |
|  | Crab × Species | 1 | 3465 | 4.71 | 0.038 | * | 1 | 11.774 | 7.963 | 0.008 | ** |
|  | Residual | 27 | 736 |  |  |  | 27 | 1.479 |  |  |  |
| Aboveground biomass | Year | 1 | 26035 |  |  |  | 1 | 1.277 |  |  |  |
|  | Crab | 1 | 509115 | 13.581 | 0.001 | ** | 1 | 0.085 | 0.071 | 0.791 |  |
|  | Species | 1 | 203828 | 5.437 | 0.027 | * | 1 | 23.805 | 19.87 | <0.001 | *** |
|  | Crab × Species | 1 | 222481 | 5.935 | 0.021 | * | 1 | 12.064 | 10.068 | 0.003 | ** |
|  | Residual | 27 | 37488 |  |  |  | 27 | 1.198 |  |  |  |

Asterisks indicate level of significance (＋ <0.1, * <0.05, ** <0.01, *** <0.001).
